# Regional changes in inpatient psychiatric bed capacity and availability of alternative psychiatric services, 2012-2022

**DOI:** 10.1093/haschl/qxaf204

**Published:** 2025-10-27

**Authors:** Michael X Liu, Emma E McGinty, William L Schpero

**Affiliations:** Division of Health Policy and Economics, Department of Population Health Sciences, Weill Cornell Medical College, New York, NY 10022, United States; Division of Health Policy and Economics, Department of Population Health Sciences, Weill Cornell Medical College, New York, NY 10022, United States; Cornell Health Policy Center, Cornell University, New York, NY 10022, United States; Division of Health Policy and Economics, Department of Population Health Sciences, Weill Cornell Medical College, New York, NY 10022, United States; Cornell Health Policy Center, Cornell University, New York, NY 10022, United States

**Keywords:** inpatient psychiatric care, psychiatric bed shortage, mental health services

## Abstract

**Introduction:**

The US faces a growing mismatch between demand for inpatient psychiatric care and available capacity. Little is known about the characteristics of regions affected by inpatient psychiatric bed shortages, which hospitals have faced decreases in bed supply, and whether other psychiatric services have emerged to fill the gap.

**Methods:**

Using data from the American Hospital Association Annual Survey, we conducted a descriptive analysis of inpatient psychiatric bed supply across hospital referral regions (HRRs) from 2012 to 2022. We examined the demographic patterns of regions affected by shortages, assessed hospital characteristics associated with reductions in psychiatric capacity, and evaluated the presence of alternative psychiatric services that may substitute for inpatient care.

**Results:**

More than 60% of the US population consistently lived in HRRs with psychiatric bed shortages during this period, defined as fewer than 30 beds per 100 000 people. By 2022, HRRs with severe shortages, relative to those without them, were more likely to be in the West and had higher proportions of Hispanic residents, raising concerns about inequities in behavioral health care access. Hospitals most likely to reduce psychiatric capacity were general, non-profit, and system-affiliated institutions with lower total margins. Importantly, hospitals in severe shortage areas were less likely to have outpatient psychiatric services, indicating that alternative hospital-based resources may not fully offset inpatient shortfalls.

**Conclusion:**

Addressing the nation's psychiatric bed shortage will require targeted financial support for general hospitals at risk of closing psychiatric units and investment in broader psychiatric infrastructure to ensure equitable access across regions.

## Introduction

The US has experienced a sharp increase in demand for inpatient psychiatric care over the past decade.^1^ At the same time, the supply of inpatient psychiatric beds has mildly declined, which evidence suggests has the potential to affect psychiatric readmissions, suicide risk, and other mental health outcomes.^2,3^ Longstanding policy and financial barriers have discouraged investment in inpatient psychiatric infrastructure.^4^ These include Medicaid's Institution for Mental Diseases (IMD) exclusion, which restricts reimbursement for inpatient psychiatric care in larger facilities, as well as persistently low reimbursement rates for and high operational costs of inpatient psychiatry.^5^ In response to these constraints, some hospitals have consolidated with larger systems to offset the financial pressures of inpatient psychiatry^6^ and others have shifted toward alternative psychiatric care models, such as consultation-liaison, emergency, and outpatient psychiatric services.^7^

Efforts to increase the supply of inpatient psychiatric care have been hindered by gaps in evidence about where investment is most needed and which hospitals are most at risk of closing psychiatric units.^8^ In this study, we identified the characteristics of regions affected by inpatient psychiatric bed shortages and the characteristics of hospitals most likely to experience meaningful bed decreases. We also determined whether the availability of alternative psychiatric services may, in some cases, substitute for low access to inpatient care. The goal of this work is to inform the targeting of policy interventions to improve access to psychiatric care.

## Methods

### Data sources and variables

Data on availability of inpatient psychiatric beds for general and psychiatric hospitals came from the 2012-2022 American Hospital Association (AHA) Annual Survey, which is commonly used in studies of inpatient psychiatric bed capacity.^3^ The AHA survey also provided information on hospital-level characteristics, such as hospital size,^9^ profit status,^10^ system affiliation,^11^ and teaching status,^12^ which are proxies for hospitals’ resource capacity. In addition, the AHA survey provided information on the availability of three alternative psychiatric services that may, in some cases, substitute for inpatient care: consult-liaison psychiatry, psychiatric emergency care, and outpatient psychiatry.^7^ For example, prior work has shown comparable outcomes for patients with depression treated in the inpatient relative to outpatient settings.^13^ Additional information on hospital financial and operating characteristics came from the 2022 Centers for Medicare and Medicaid Services Healthcare Cost Report Information System dataset compiled by RAND, including total and operating margins, Medicaid share of inpatient days, and uncompensated care as a share of operating expenses. These variables are commonly used as measures of hospitals’ financial precarity; we hypothesized that financially vulnerable hospitals may have been more likely to reduce their inpatient psychiatric capacity.^14^ To address outliers, we winsorized all financial measures at the 1^st^ and 99^th^ percentiles, as has been done in prior work.^15^

We aggregated zip-code-level data from the 2012-2022 American Community Survey five-year estimates to determine area-level characteristics that capture structural barriers to accessing care, including health care coverage and race and ethnicity. These characteristics included insurance status (proportion of the population with Medicaid, private insurance, or uninsured),^16^ proportion of population in rural areas,^17^ and proportion of population that identified as Asian, Black, Hispanic, Native American, Pacific Islander, White, or other race/ethnicity.^18^

We aggregated zip-code-tabulation-area-level data from the 2022 CDC PLACES dataset to create indicators of community mental health and substance use need. Specifically, we examined the prevalence of adult binge drinking, depression, and frequent mental distress (defined as ≥14 mentally unhealthy days in the past month).

### Defining bed shortages

We examined the prevalence of bed shortages across the 306 hospital referral regions (HRRs) in the US. Recent studies have primarily examined psychiatric bed supply at the county level,^12^ yet patients frequently travel across counties and even states to access inpatient psychiatric care.^19^ We defined bed shortages using previously established criteria developed using the Delphi consensus-building method: mild (26-30 psychiatric beds per 100 000 people); moderate (15-25 psychiatric beds per 100 000 people), and severe (less than 15 psychiatric beds per 100 000 people).^20^ Optimal non-shortages were defined as greater than 60 beds per 100 000 people and sufficient non-shortages were 31 to 60 beds per 100 000 people.^20^

### Statistical analysis

We first examined differences in the characteristics of HRRs facing severe shortages relative to those facing mild, moderate, or no shortages using t-tests (for continuous variables) and tests of proportions (for categorical variables). To compare HRRs and hospitals with the largest increases vs largest decreases in bed supply over the study period, we compared the characteristics of HRRs and hospitals, respectively, with above median percent increases in bed supply with HRRs and hospitals with below median percent decreases in bed supply. Lastly, we compared the proportion of hospitals offering each of the three alternative services in HRRs with severe shortages relative to those with no shortages.

The institutional review board at Weill Cornell Medical College deemed this study not human subjects research. All analyses were performed using Stata 16.1 (StataCorp). Statistical significance was determined at *P* < 0.05.

### Limitations

Our study has several limitations. First, 19.2% of hospital-years had missing psychiatric bed data, which we imputed using a conservative imputation method based on established approaches to preserve sample size (see Appendix Table SA1).^21^ Missing data on alternative psychiatric services in 2022 (24.2% of hospitals) were imputed using the same approach.^21^ We tested the sensitivity of our analysis using non-imputed data and found similar results (see Appendix Figure SA1). Second, psychiatric bed counts and alternative psychiatric services were hospital-reported in the AHA annual survey, which may introduce measurement error. The AHA survey, however, has generally been viewed as a valid source of data on psychiatric bed supply and has commonly been used for this purpose in the literature.^3^ Third, AHA data on psychiatric bed availability do not differentiate between types of psychiatric beds (eg, general vs specialty); therefore, it may not be accurate to assume that all beds should be treated as equally affecting available supply. Fourth, our analysis relied on HRRs to define local markets. While HRRs capture cross-county patient flows,^19^ they were originally constructed from a Medicare population and may not perfectly reflect the catchment areas for psychiatric care. Finally, alternative psychiatric services in our data were limited to hospital-reported consult-liaison, outpatient, and psychiatric emergency services, and did not include community-based programs offered outside hospitals. More granular data on service availability and patient experiences of access will be important for future work.

## Results

Our sample included 4067 general and psychiatric hospitals across 306 hospital referral regions (HRRs) between 2012 and 2022 (see Appendix Figure SA2). In 2012, 60.7% of the US population lived in areas classified as having inpatient psychiatric bed shortages; by 2022, this figure remained largely unchanged at 64.9% (see Appendix Figure SA3). Among the 306 HRRs, 61.1% were categorized as experiencing shortages in 2012, compared with 62.7% in 2022 (see Appendix Figure SA4).

**Figure 1.**
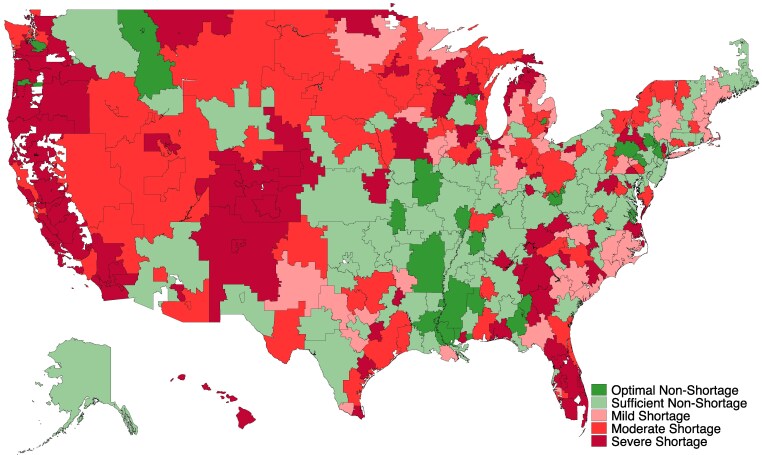
Inpatient psychiatric bed shortage status across hospital referral regions, 2022. Source/Notes: SOURCE Authors’ analysis of data from the 2022 American Hospital Association Annual Survey and the 2022 American Community Survey five-year estimates. NOTES Inpatient psychiatric bed shortages were defined by the Delphi criteria,^7^ where severe shortages are 14 or less inpatient psychiatric beds per 100 000 people, moderate shortages are 15 to 25 inpatient psychiatric beds per 100 000 people, mild shortages are 26 to 30 inpatient psychiatric beds per 100 000 people, sufficient non-shortages are 31 to 59 inpatient psychiatric beds per 100 000 people, and optimal non-shortages are 60 inpatient psychiatric beds or greater per 100 000 people.

In 2022, HRRs with severe shortages were more likely to be in the West compared with HRRs without shortages (40.4% vs 11.1%, *P* < 0.01) (Figure 1). HRRs with severe shortages had a higher proportion of the population that identified as Hispanic (19.8% vs 12.3%, *P* < 0.01), while a lower proportion of the population identified as Black (10.4% vs 13.4%, *P* = 0.04), compared to HRRs with no, mild, or moderate shortages (Table 1). HRRs with severe shortages did not differ in terms of prevalence of binge drinking, depression, or frequent mental distress when compared to HRRs with no, mild, or moderate shortages.

**Table 1.** Characteristics of hospital referral regions by inpatient psychiatric bed shortage status, 2022.

	No/Mild/Moderate shortage	Severe shortage	Mean difference (95% CI)	*P*-value
Number of HRRs	217	89		
Inpatient Psychiatric Beds per 100 000 people, Mean N (SD)	37.7 (22.1)	8.8 (4.5)	−28.9 (−33.6 to −24.2)	<0.01
Insurance, Mean % (SD)				
Medicaid	20.0 (5.4)	21.7 (6.0)	1.7 (0.3 to 3.1)	0.01
Private	66.4 (7.1)	64.7 (7.4)	−1.7 (−3.5 to 0.1)	0.1
Uninsured	8.3 (3.9)	8.3 (3.6)	0.0 (−0.9 to 0.9)	1.0
Race and Ethnicity, Mean % (SD)				
Asian	4.4 (5.1)	5.7 (8.0)	1.3 (−0.2 to 2.8)	0.1
Black	13.4 (11.2)	10.4 (10.8)	−2.9 (−5.7 to −0.2)	0.04
Hispanic	12.3 (13.3)	19.8 (17.8)	7.4 (3.8 to 11.1)	<0.01
Native American	2.1 (2.6)	2.5 (2.7)	0.4 (−0.2 to 1.1)	0.2
Other	7.7 (7.2)	11.8 (10.1)	4.1 (2.1 to 6.1)	<0.01
Pacific Islander	0.3 (0.4)	0.7 (2.8)	0.4 (0.0 to 0.8)	0.04
White	79.5 (13.1)	79.0 (14.0)	−0.5 (−3.8 to 2.8)	0.8
Region, N (%)				
Midwest	69 (31.8)	20 (22.5)	−9.3 (−20.0 to 1.3)	0.1
Northeast	38 (17.5)	5 (5.6)	−11.9 (−18.9 to −4.9)	<0.01
South	86 (39.6)	28 (31.5)	−8.2 (−19.8 to 3.5)	0.2
West	24 (11.1)	36 (40.4)	29.4 (18.4 to 40.4)	<0.01
Rural, Mean % (SD)	29.5 (24.9)	24.5 (24.0)	−5.1 (−11.2 to 1.0)	0.1
Mental Health, Mean % (SD)				
Binge Drinking	17.0 (2.3)	17.0 (2.0)	0.0 (−0.5 to 0.5)	1.0
Depression	23.2 (3.1)	23.1 (3.2)	−0.1 (−0.9 to 0.7)	0.8
Frequent Mental Health Distress	17.6 (2.0)	17.6 (1.7)	−0.0 (−0.5 to 0.5)	1.0

Source/Notes: SOURCE Authors’ analysis of data from the 2022 American Hospital Association Annual Survey, 2010-2022 American Community Survey five-year estimates, and 2022 Center for Disease Control and Prevention PLACES dataset. NOTES Inpatient psychiatric bed shortages were defined by Delphi criteria,^7^ where no/mild/moderate shortages are characterized as HRRs with inpatient psychiatric bed capacity of 15 beds or greater per 100 000 people, while severe shortages are HRRs with inpatient psychiatric bed capacity of 14 or fewer beds per 100 000 people. Mean inpatient psychiatric bed capacity is reported per 100 000 people.

HRRs with the largest decreases in inpatient psychiatric bed capacity 2012-2022 (mean decrease −41.0%), when compared to HRRs that had largest increases (mean increase 77.5%), did not differ in race/ethnicity and insurance composition, region, or rurality (see Appendix Table SA2). HRRs with the largest decreases also did not differ in prevalence of depression, binge drinking, or frequent mental health distress in 2022 when compared with HRRs with the largest increases.

Hospitals that experienced the largest decreases in inpatient psychiatric bed capacity (mean decrease −83.3%), compared to those with the largest increases (mean increase 110.2%), were more likely to be general medical (95.7% vs 77.7%, *P* < 0.01), non-profit (71.5% vs 50.3%, *P* < 0.01) hospitals, and a member of a health system (55.1% vs 46.5%, *P* = 0.04) (Table 2). Financially, hospitals with the largest decreases in inpatient psychiatric bed capacity had lower total margins (−1.8% vs 1.4%, *P* < 0.01).

**Table 2.** Characteristics of hospitals by change in inpatient psychiatric bed supply, 2012-2022.

	Large decrease	Small decrease	No change	Small increase	Large increase	Mean difference, large decrease vs large increase (95% CI)	*P*-value
Number of Hospitals	256	257	3482	302	310		
Change in Inpatient Bed Capacity, Mean % (SD)	−83.3 (24.0)	−16.6 (10.0)	0.0 (0.0)	20.0 (13.7)	110.2 (68.5)	−193.5 (−202.3 to −184.7)	< 0.01
Hospital Type, N (%)							
General Medical	245 (95.7)	186 (72.4)	3270 (93.9)	186 (61.6)	241 (77.7)	18.0 (12.7 to 23.2)	< 0.01
Psychiatric	11 (4.3)	71 (27.6)	212 (6.1)	116 (38.4)	69 (22.3)	−17.5 (−22.5 to −12.5)	< 0.01
Profit Status, N (%)							
For-Profit	35 (13.7)	37 (14.4)	525 (15.1)	87 (28.8)	93 (30.0)	−16.3 (−22.9 to −9.7)	< 0.01
Government	38 (14.8)	65 (25.3)	831 (23.9)	46 (15.2)	61 (19.7)	−4.8 (−11.0 to 1.4)	0.1
Non-Profit	183 (71.5)	155 (60.3)	2126 (61.1)	169 (56.0)	156 (50.3)	21.2 (13.3 to 29.0)	< 0.01
Size, N (%)							
Less than 100 Beds	72 (28.1)	52 (20.2)	2138 (61.4)	59 (19.5)	91 (29.4)	−1.2 (−8.7 to 6.3)	0.7
100 to 300 Beds	101 (39.5)	112 (43.6)	941 (27.0)	136 (45.0)	135 (43.5)	−4.1 (−12.2 to 4.0)	0.3
Greater than 300 Beds	83 (32.4)	93 (36.2)	403 (11.6)	107 (35.4)	84 (27.1)	5.3 (−2.2 to 12.9)	0.2
System Affiliation, N (%)	141 (55.1)	121 (47.1)	1455 (41.8)	158 (52.3)	144 (46.5)	8.6 (0.4 to 16.9)	0.04
Teaching Hospital, N (%)	30 (11.7)	41 (16.0)	98 (2.8)	30 (9.9)	29 (9.4)	2.4 (−2.7 to 7.5)	0.4
Medicaid Share, Mean % (SD)	24.1 (10.5)	26.7 (14.1)	17.7 (13.7)	27.7 (16.0)	25.6 (16.4)	−1.5 (−3.9 to 0.9)	0.2
Operating Margin, Mean % (SD)	−2.8 (15.3)	−5.2 (23.9)	−0.2 (17.2)	−2.5 (21.8)	−1.1 (17.4)	−1.6 (−4.4 to 1.1)	0.2
Total Margin, Mean % (SD)	−1.8 (12.9)	−1.1 (14.4)	2.0 (13.4)	0.3 (15.2)	0.8 (12.9)	−2.6 (−4.8 to −0.4)	0.02
Uncompensated Care Share, Mean % (SD)	6.8 (4.1)	7.3 (6.3)	7.5 (5.4)	6.3 (4.4)	6.6 (5.3)	0.2 (−0.7 to 1.0)	0.7

SOURCE Authors’ analysis of data from the 2012-2022 American Hospital Association Annual Survey and the 2012-2022 Healthcare Cost Report Information System. NOTES Hospital referral region inpatient psychiatric bed capacity percent changes were created where large decreases represent the bottom half of negative percent change from 2012 to 2022, and large increases represent the top half of positive percent change from 2012 to 2022. Medicaid share was defined as share of inpatient days and uncompensated care share represents uncompensated care costs as a percent of total operating expenditures.

**Figure 2.**
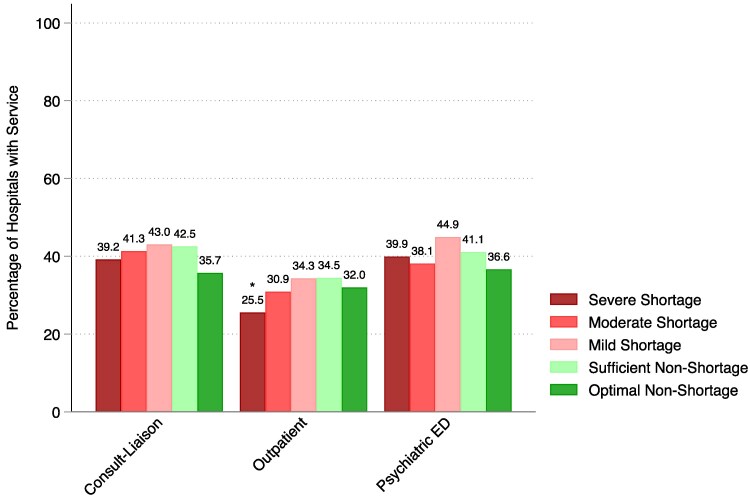
Availability of alternative psychiatric services by hospital referral region inpatient psychiatric bed shortage status, 2022. Source/Notes: SOURCE Authors’ analysis of data from the 2022 American Hospital Association Annual Survey and the 2022 American Community Survey five-year estimates. NOTES Inpatient psychiatric bed shortages were defined by the Delphi criteria,^7^ where severe shortages are 14 or less inpatient psychiatric beds per 100 000 people, moderate shortages are 15 to 25 inpatient psychiatric beds per 100 000 people, mild shortages are 26 to 30 inpatient psychiatric beds per 100 000 people, sufficient non-shortages are 31 to 59 inpatient psychiatric beds per 100 000 people, and optimal non-shortages are 60 inpatient psychiatric beds or greater per 100 000 people.

HRRs with severe shortages were less likely to have outpatient psychiatric services (25.5% vs 34.4%, *P* < 0.01) than those with no shortages (Figure 2). Sensitivity analyses without imputation of missing alternative psychiatric services data showed similar trends (Appendix Figure SA1).

## Conclusion

In this study, we found that more than 60% of the US population consistently lived in a region with an inpatient psychiatric bed shortage over 2012-2022, underscoring the persistence and scale of the bed supply crisis. Our study aligns with recent work documenting mildly declining numbers of inpatient psychiatric beds nationally, driven primarily by bed closures in general hospitals.^3^ We extend this evidence by translating changes in supply into area-level indicators of bed shortages, highlighting which parts of the country require targeted interventions.

Shortages were not evenly distributed, but instead disproportionately affected certain populations and regions. Severe psychiatric bed shortages in 2022 occurred in areas that had higher proportions of residents who identified as Hispanic, suggesting that psychiatric bed scarcity may disproportionately affect certain communities. Hispanic populations already face lower rates of depression treatment, contributing to greater chronicity and functional impairment.^18^ Psychiatric bed shortages in these regions may therefore magnify existing inequities and widen gaps in mental health outcomes.

Our analysis further highlights the structural and financial pressures facing the 11.1% of hospitals that experienced decreases in psychiatric bed capacity. Those hospitals that experienced the largest decreases had significantly lower total, but not operating margins, indicating that hospitals with decreased psychiatric bed capacity often face broader financial pressures beyond the direct cost of providing care. Such pressures may include the capital burden of required infrastructure upgrades, staffing needs, and uncompensated care, which can be prohibitively expensive for financially vulnerable hospitals.^22^

Additionally, hospitals that reduced psychiatric bed capacity were more often affiliated with health systems, which is concerning given that the proportion of community hospitals within larger systems rose from 53% in 2005% to 68% in 2022.^3^ This pattern suggests that growing consolidation may further hinder access to inpatient psychiatric care.

We also found that shortages in inpatient capacity were not offset by greater availability of alternative psychiatric services. Hospitals in regions with the most severe psychiatric bed shortages were less likely to offer outpatient psychiatric services, underscoring how inpatient psychiatric bed shortages may not be compensated for by outpatient capacity. Also, over half of hospitals in these high-shortage regions lacked any of the alternative psychiatric services we examined, highlighting a critical gap in care when both inpatient and substitute models are often unavailable or insufficient to meet demand. Community-based services not captured in this study's data may address some of these gaps, but shortages in community mental health services are also widespread.^23^

Taken together, these findings have important policy implications. Evidence suggests that Section 1115 Medicaid waivers, which some states have used to fund Medicaid services in IMDs, have modestly expanded access to psychiatric care and treatment options.^10,12,24^ The IMD exclusion therefore still constrains access to specialized inpatient psychiatric care, shifting pressure onto general medical hospitals,^2^ facilities which we found in our study to be more likely to reduce psychiatric bed capacity. These findings underscore the need to consider a constellation of policies, including Medicaid reform that moves beyond temporary Section 1115 waivers, in addition to expanding access to inpatient psychiatric care across all hospital facility types.

Addressing gaps in inpatient bed capacity will require targeting investments to underserved areas and communities with high psychiatric need, enhancing financial support to at-risk hospitals, advancing Medicaid reforms, and strengthening the availability of non-inpatient psychiatric services in hospitals and the community.

Finally, our study highlights the need for more granular and patient-centered measures of psychiatric care access. Administrative surveys such as the AHA Annual Survey do not distinguish between bed types or capture barriers such as insurance acceptance and referral requirements. In addition, bed-to-population ratios do not take into account true demand for inpatient care, although we notably found no differences in rates of depression, binge drinking, or frequent mental distress across shortage and non-shortage HRRs. Future research should integrate measures of community mental health to better align definitions of shortage with actual patterns of need.

Ensuring equitable and sustainable access to inpatient psychiatric care is a critical component of addressing the broader U.S. mental health crisis.

## Supplementary Material

qxaf204_Supplementary_Data
