# Supplementary material for: Regional changes in inpatient psychiatric bed capacity and availability of alternative psychiatric services, 2012-2022
Source: Health Aff Sch. 2025 Oct 27;3(11):qxaf204. doi: 10.1093/haschl/qxaf204 (PMC12610395; doi:10.1093/haschl/qxaf204)
Supplement: qxaf204_Supplementary_Data [file qxaf204_supplementary_data.zip › Supplement_final.docx]

**Online Appendix**

**Regional Changes in Inpatient Psychiatric Bed Capacity and Availability of Alternative Psychiatric Services, 2012-2022**

PP2: Technical Appendix, Imputation

PP3: Table A1: Inpatient psychiatric bed data missingness by year before and after imputation

PP4-5: Table A2: Characteristics of hospital referral regions by change in per capita inpatient psychiatric bed supply, 2012–2022

PP6: Figure A1: Availability of non-imputed alternative psychiatric services by hospital referral region inpatient psychiatric bed shortage status, 2022

PP7: Figure A2: Construction of the analytical sample

PP8: Figure A3: Proportion of population residing in hospital referral regions with inpatient psychiatric bed shortages, 2012-2022

PP9: Figure A4: Proportion of hospital referral regions with inpatient psychiatric bed shortages, 2012 to 2022

**Technical Appendix: Imputation**

**Handling Missing Bed Counts**

Psychiatric bed counts were missing in 19.2% of hospital-year observations, with increasing missingness in 2020–2022. We used a conservative imputation strategy based on prior literature:^8^

1. We carried forward the most recent non-missing value or carried backward from the first available value.

This approach allowed retention of longitudinal trends while minimizing bias from selective reporting.

**Handling Missing Alternative Psychiatric Service Counts**

Alternative psychiatric service counts were missing in 24.2% of hospital observations in 2022. We used a conservative imputation strategy based on prior literature:^8^

1. We carried forward the most recent non-missing value to 2022.

Services that had less than 10% missingness were included for analysis. For sensitivity analysis without imputation, see Appendix Figure A1.

Table A1: Inpatient psychiatric bed data missingness by year before and after imputation

| Year | Hospitals with Missing Psychiatric Bed Data Before Imputation (%) | Hospitals with Missing Psychiatric Bed Data After Imputation (%) |
| --- | --- | --- |
| 2012 | 13.9 | 0.2 |
| 2013 | 13.9 | 0.1 |
| 2014 | 16.5 | 0.1 |
| 2015 | 14.0 | 0.1 |
| 2016 | 16.8 | 0.1 |
| 2017 | 21.6 | 0.1 |
| 2018 | 19.3 | 0.1 |
| 2019 | 22.2 | 0.1 |
| 2020 | 24.0 | 0.1 |
| 2021 | 24.5 | 0.1 |
| 2022 | 24.0 | 0.1 |

Table A2: Characteristics of hospital referral regions by change in per capita inpatient psychiatric bed supply, 2012–2022

|  | Large Decrease | Small Decrease | No Change | Small Increase | Large Increase | Mean Difference, Large Decrease vs Large Increase  (95% CI) | P-value |
| --- | --- | --- | --- | --- | --- | --- | --- |
| Number of HRRs | 73 | 74 | 3 | 78 | 78 |  |  |
| Inpatient Psychiatric Beds per 100,000 people, Mean N (SD) | 18.9 (16.1) | 32.0 (28.2) | 0.0 (0.0) | 32.9 (19.0) | 33.9 (23.3) | -15.0 (-21.5 to -8.5) | < 0.01 |
| Change in Inpatient Psychiatric Bed Capacity, Mean % (SD) | -41.0 (25.7) | -7.3 (4.1) | 0.0 (0.0) | 8.4 (5.3) | 77.5 (121.9) | -118.4 (-147.2 to -89.7) | < 0.01 |
| Insurance, Mean % (SD) |  |  |  |  |  |  |  |
| Medicaid | 21.2 (5.4) | 21.1 (6.8) | 16.1 (2.8) | 19.5 (4.7) | 20.3 (5.4) | 0.8 (-0.9 to 2.6) | 0.3 |
| Private | 65.2 (8.2) | 65.9 (7.4) | 71.2 (2.0) | 67.1 (6.1) | 65.1 (7.0) | 0.1 (-2.3 to 2.6) | 0.9 |
| Uninsured | 8.6 (4.5) | 7.7 (3.8) | 8.2 (4.1) | 8.2 (3.2) | 8.8 (3.6) | -0.3 (-1.6 to 1.0) | 0.7 |
| Race and Ethnicity, Mean % (SD) |  |  |  |  |  |  |  |
| Asian | 4.0 (4.6) | 5.0 (4.6) | 5.8 (0.8) | 4.7 (5.0) | 5.3 (9.0) | -1.2 (-3.6 to 1.1) | 0.3 |
| Black | 12.4 (12.1) | 11.5 (10.0) | 6.0 (6.1) | 13.7 (10.8) | 12.5 (11.7) | -0.1 (-3.9 to 3.7) | 1.0 |
| Hispanic | 16.0 (17.1) | 14.7 (16.8) | 25.0 (3.2) | 13.8 (13.5) | 13.2 (13.2) | 2.9 (-2.0 to 7.7) | 0.3 |
| Native American | 2.1 (2.5) | 2.0 (1.8) | 2.4 (0.8) | 2.2 (2.8) | 2.6 (3.2) | -0.5 (-1.4 to 0.4) | 0.3 |
| Other | 9.4 (8.4) | 9.1 (9.5) | 14.5 (5.3) | 8.8 (8.5) | 7.9 (6.9) | 1.5 (-0.9 to 4.0) | 0.2 |
| Pacific Islander | 0.3 (0.3) | 0.4 (0.4) | 0.5 (0.4) | 0.3 (0.4) | 0.6 (3.0) | -0.4 (-1.1 to 0.3) | 0.3 |
| White | 79.9 (13.9) | 80.2 (13.6) | 82.1 (5.8) | 78.2 (12.5) | 79.1 (13.7) | 0.8 (-3.6 to 5.3) | 0.7 |
| Region, N (%) |  |  |  |  |  |  |  |
| Midwest | 22 (30.1) | 22 (29.7) | 0 (0.0) | 26 (33.3) | 19 (24.4) | 5.8 (-8.4 to 20.0) | 0.4 |
| Northeast | 13 (17.8) | 15 (20.3) | 0 (0.0) | 9 (11.5) | 6 (7.7) | 10.1 (-0.5 to 20.7) | 0.1 |
| South | 24 (32.9) | 22 (29.7) | 1 (33.3) | 30 (38.5) | 37 (47.4) | -14.6 (-30.0 to 0.9) | 0.1 |
| West | 14 (19.2) | 15 (20.3) | 2 (66.7) | 13 (16.7) | 16 (20.5) | -1.3 (-14.1 to 11.4) | 0.8 |
| Rural, Mean % (SD) | 27.8 (23.4) | 27.0 (23.5) | 13.9 (12.6) | 25.8 (23.3) | 32.1 (28.5) | -4.3 (-12.7 to 4.1) | 0.3 |
| Mental Health, Mean % (SD) |  |  |  |  |  |  |  |
| Binge Drinking | 17.1 (2.4) | 17.1 (1.9) | 18.6 (0.7) | 17.1 (2.1) | 16.8 (2.4) | 0.3 (-0.4 to 1.1) | 0.4 |
| Depression | 23.0 (3.3) | 23.0 (2.9) | 23.5 (1.9) | 23.4 (2.7) | 23.4 (3.7) | -0.4 (-1.5 to 0.8) | 0.5 |
| Frequent Mental Health Distress | 17.7 (2.2) | 17.5 (1.6) | 17.5 (1.7) | 17.5 (1.6) | 17.6 (2.3) | 0.1 (-0.6 to 0.8) | 0.8 |

Source/Notes: SOURCE Authors’ analysis of data from the 2012-2022 American Hospital Association Annual Survey, 2010-2022 American Community Survey five-year estimates, and 2022 Center for Disease Control and Prevention PLACES dataset. NOTES Hospital referral region inpatient psychiatric bed capacity percent changes were created where large decreases represent the bottom half of negative percent change from 2012 to 2022, and large increases represent the top half of positive percent change from 2012 to 2022. Mean inpatient psychiatric bed capacity is reported per 100,000 people

Figure A1: Availability of non-imputed alternative psychiatric services by hospital referral region inpatient psychiatric bed shortage status, 2022


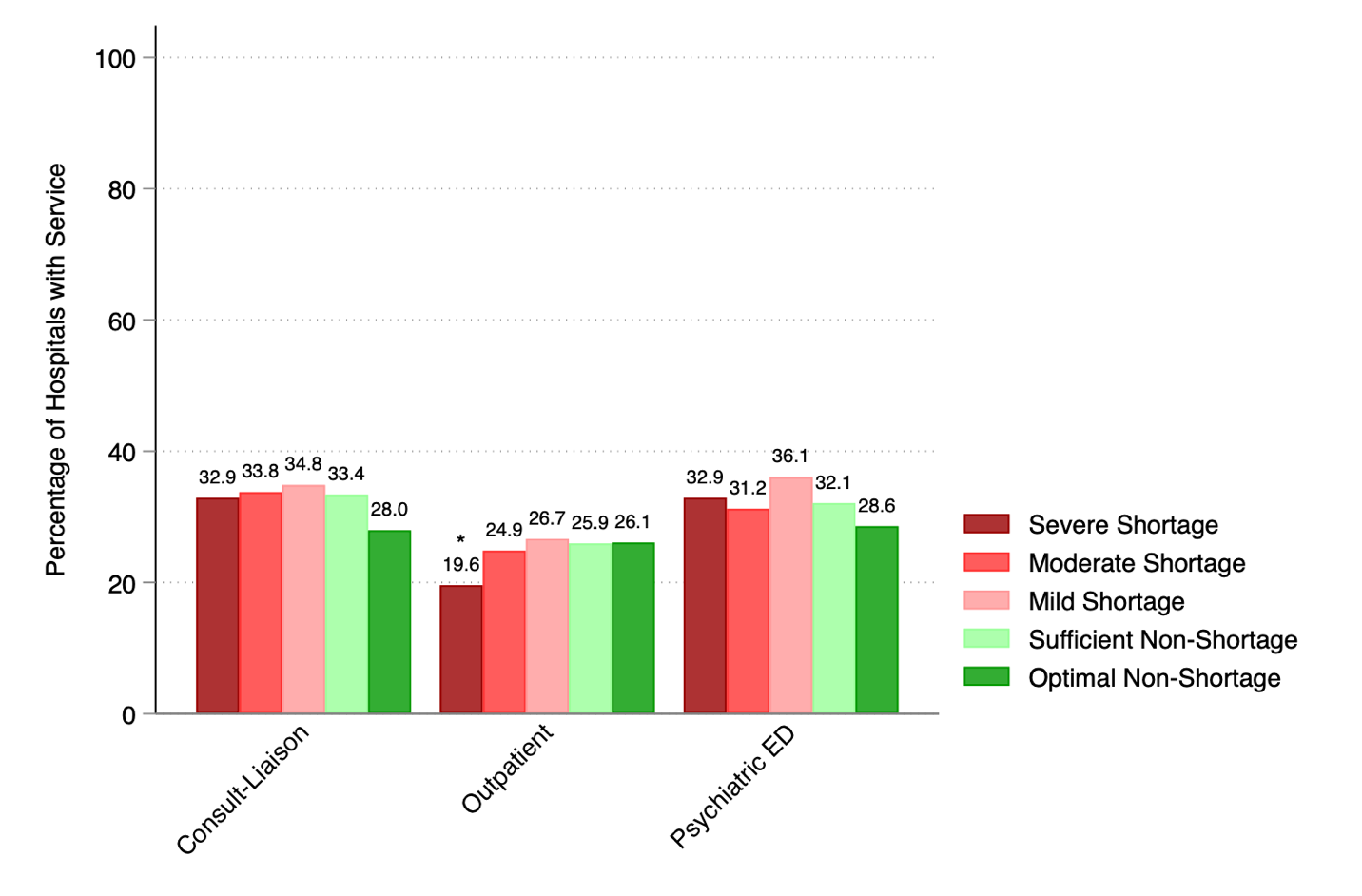


SOURCE Authors’ analysis of data from the 2022 American Hospital Association Annual Survey and the 2022 American Community Survey five-year estimates. NOTES Inpatient psychiatric bed shortages were defined by the Delphi criteria,^7^ where severe shortages are 14 or less inpatient psychiatric beds per 100,000 people, moderate shortages are 15 to 25 inpatient psychiatric beds per 100,000 people, mild shortages are 26 to 30 inpatient psychiatric beds per 100,000 people, sufficient non-shortages are 31 to 59 inpatient psychiatric beds per 100,000 people, and optimal non-shortages are 60 inpatient psychiatric beds or greater per 100,000 people. *p < 0.05

Figure A2: Construction of analytic sample


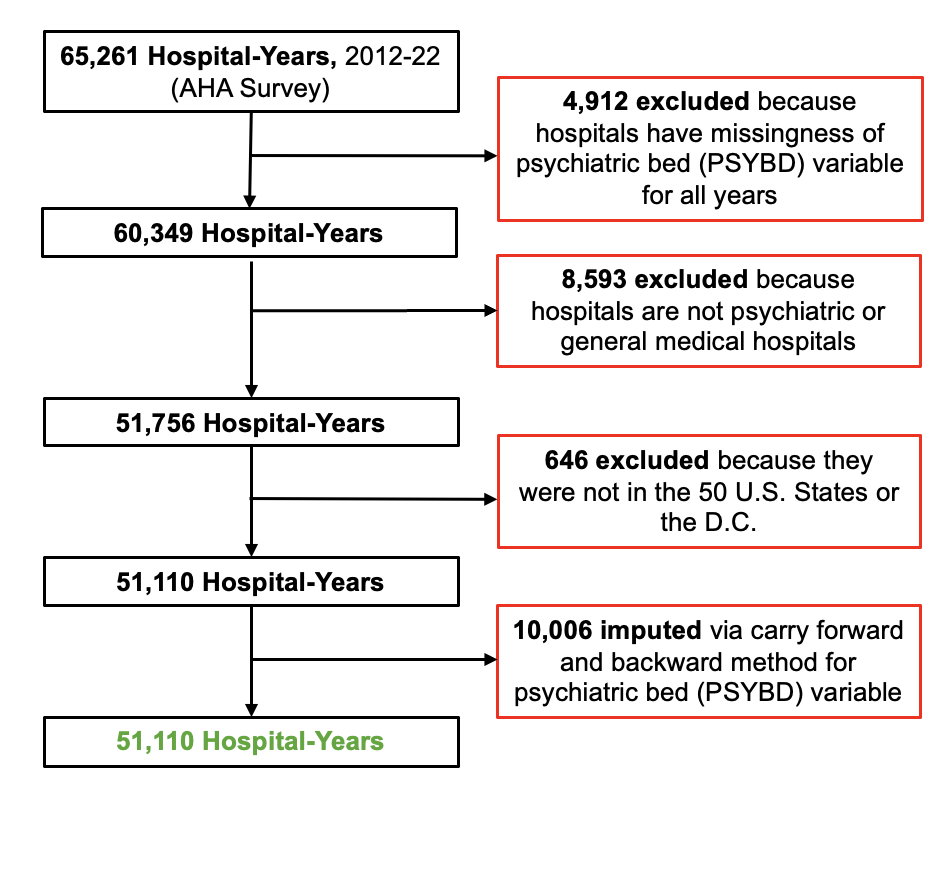


Figure A3: Proportion of population residing in hospital referral regions with inpatient psychiatric bed shortages, 2012-2022


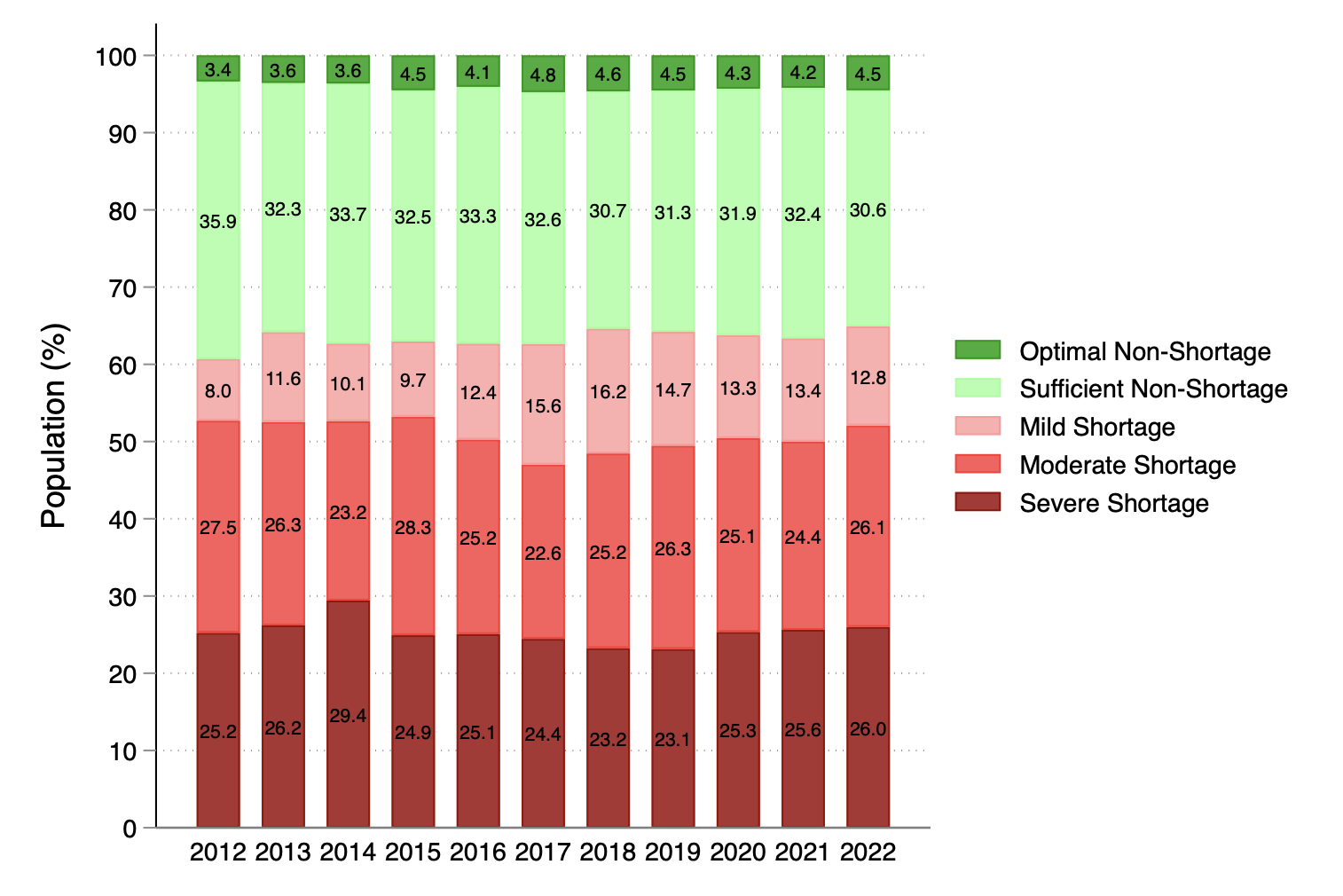


SOURCE Authors’ analysis of data from the 2012-2022 American Hospital Association Annual Survey and the 2012-2022 American Community Survey five-year estimates. NOTES Inpatient psychiatric bed shortages were defined by the Delphi criteria,^7^ where severe shortages are 14 or less inpatient psychiatric beds per 100,000 people, moderate shortages are 15 to 25 inpatient psychiatric beds per 100,000 people, mild shortages are 26 to 30 inpatient psychiatric beds per 100,000 people, sufficient non-shortages are 31 to 59 inpatient psychiatric beds per 100,000 people, and optimal non-shortages are 60 inpatient psychiatric beds or greater per 100,000 people.

Figure A4: Proportion of hospital referral regions with inpatient psychiatric bed shortages, 2012-2022


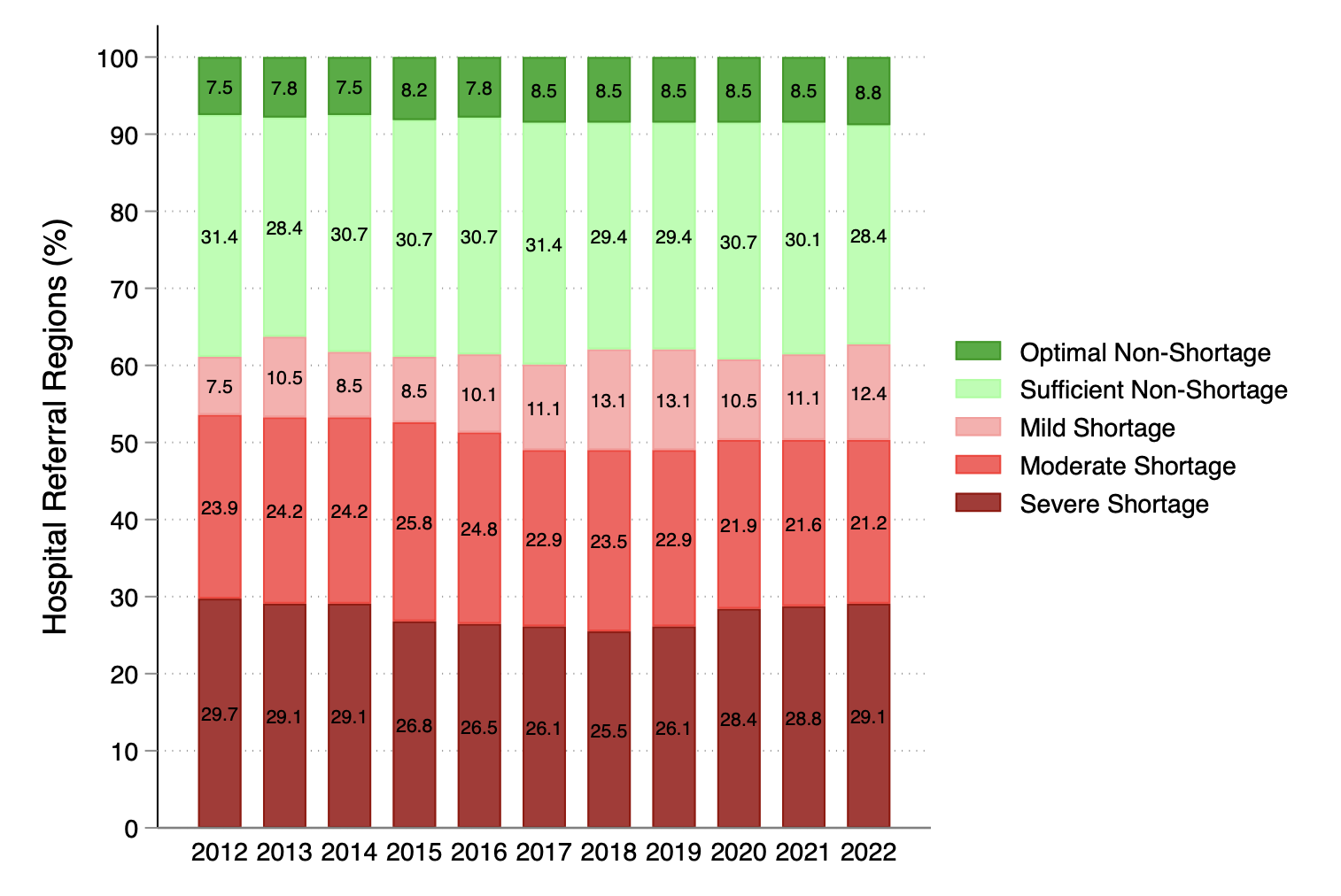


SOURCE Authors’ analysis of data from the 2012-2022 American Hospital Association Annual Survey and the 2012-2022 American Community Survey five-year estimates. NOTES Inpatient psychiatric bed shortages were defined by the Delphi criteria,^7^ where severe shortages are 14 or less inpatient psychiatric beds per 100,000 people, moderate shortages are 15 to 25 inpatient psychiatric beds per 100,000 people, mild shortages are 26 to 30 inpatient psychiatric beds per 100,000 people, sufficient non-shortages are 31 to 60 inpatient psychiatric beds per 100,000 people, and optimal non-shortages are 61 inpatient psychiatric beds or greater per 100,000 people.
